# Epidemiology of malaria in the forest-savanna transitional zone of Ghana

**DOI:** 10.1186/1475-2875-8-220

**Published:** 2009-09-28

**Authors:** Seth Owusu-Agyei, Kwaku Poku Asante, Martin Adjuik, George Adjei, Elizabeth Awini, Mohammed Adams, Sam Newton, David Dosoo, Dominic Dery, Akua Agyeman-Budu, John Gyapong, Brian Greenwood, Daniel Chandramohan

**Affiliations:** 1 Kintampo Health Research Centre, Kintampo, Ghana; 2 DCVB/ITD, London School of Hygiene & Tropical Medicine, UK; 3 Navrongo Health Research Centre, Navrongo, Ghana; 4 Dodowa Health Research Centre, Dodowa, Ghana; 5 Health Research Unit, Ghana Health Service, Ghana

## Abstract

**Background:**

Information on the epidemiology of malaria is essential for designing and interpreting results of clinical trials of drugs, vaccines and other interventions. As a background to the establishment of a site for anti-malarial drugs and vaccine trials, the epidemiology of malaria in a rural site in central Ghana was investigated.

**Methods:**

Active surveillance of clinical malaria was carried out in a cohort of children below five years of age (n = 335) and the prevalence of malaria was estimated in a cohort of subjects of all ages (n = 1484) over a 12-month period. Participants were sampled from clusters drawn around sixteen index houses randomly selected from a total of about 22,000 houses within the study area. The child cohort was visited thrice weekly to screen for any illness and a blood slide was taken if a child had a history of fever or a temperature greater than or equal to 37.5 degree Celsius. The all-age cohort was screened for malaria once every eight weeks over a 12-month period. Estimation of Entomological Inoculation Rate (EIR) and characterization of *Anopheline *malaria vectors in the study area were also carried out.

**Results:**

The average parasite prevalence in the all age cohort was 58% (95% CI: 56.9, 59.4). In children below five years of age, the average prevalence was 64% (95% CI: 61.9, 66.0). Geometric mean parasite densities decreased significantly with increasing age. More than 50% of all children less than 10 years of age were anaemic. Children less than 5 years of age had as many as seven malaria attacks per child per year. The attack rates decreased significantly with increasing cut-offs of parasite density. The average Multiplicity of Infection (MOI) was of 6.1. All three pyrimethamine resistance mutant alleles of the *Plasmodium falciparum dhfr *gene were prevalent in this population and 25% of infections had a fourth mutant of *pfdhps*-A437G. The main vectors were *Anopheles funestus *and *Anopheles gambiae *and the EIR was 269 infective bites per person per year.

**Conclusion:**

The transmission of malaria in the forest-savanna region of central Ghana is high and perennial and this is an appropriate site for conducting clinical trials of anti-malarial drugs and vaccines.

## Background

Over 500 million episodes of malaria occur yearly, predominantly in sub-Saharan African children under five years of age, resulting in the death of approximately a million of these children [1-3]. Severe anaemia due to malaria occurs in 1.5 to 6.0 million African children per year with a case fatality rate of about 10%; respiratory distress, hypoglycaemia and overlapping conditions contribute a further 1-2 million cases with a mortality of nearly 20% [4]. In Ghana, malaria has been [5], and remains the first cause of loss of days of healthy life [6] as it accounts for at least 20% of child deaths, 40% of child hospital admissions and more than 50% of out-patient attendances [6].

Knowledge of the epidemiology of malaria is essential for the design and interpretation of the results of trials of drugs, vaccines and other anti-malarial interventions [7-10]. Information required includes data on parasite prevalence and parasite genetic characteristics, the dynamics of the infection, attack rates, antigenic variability, the nature, behaviour and genetic characteristics of the local malaria vectors and information on patterns of morbidity from malaria. Prior to the conduct of a series of drug and vaccine trials, a study of the epidemiology of malaria were carried out in a forest area of central Ghana, an area where a few malaria studies have been done in recent years.

## Methods

### Study Site

Kintampo North and South districts lie within the forest-savanna, transitional ecological zone in the Brong Ahafo Region of Ghana. The districts have an area of 7,162 km^2 ^and a population of approximately 150,000 living in 97 communities. The mean monthly temperature ranges between 18°C and 38°C and the average rainfall is 1250 mm per annum, occurring mainly between May and October each year.

### Study participants selection

Three hundred and thirty-five children below five years of age (child cohort) and 1,484 participants of all ages (all-age cohort) from the study areas were enrolled. They came from sixteen "index houses" selected randomly from the 22,000 houses within the study area that are registered in a database of an ongoing health and demographic surveillance system. The houses were stratified initially into three micro-ecological areas (urban, rural savanna and forest) and weighted such that the number of houses selected from each of these areas was proportional to the target population in each stratum and that only one "index house" was selected from each village. From each "index house", all children less than 10 years of age and a weighted number of 10-19 years and above 19+ years old adults were selected. If an "index house" did not have the required number of participants per age group, nearby houses were visited in order of proximity to the "index house" until the required number for each age category was obtained. An "index cluster", our unit of sampling, formed the cluster of contiguous houses in which recruitment took place. An average of 87 houses represented a cluster. In total, there were 1113 participants in the age group less than 10 years weighted in each cluster, 167 in the 10-19 years age group and 204 participants in the 19+ years age-group. Study participants were recruited in November and December 2003 and followed up over a 12-month period.

### Active surveillance for incidence of clinical malaria

The child cohort was visited at home thrice weekly to check the axillary temperature and to enquire about any recent illness. If a child had an axillary temperature ≥ 37.5°C or a history of fever within the past 48 hours as reported by the caregivers, the symptoms and signs of the illness were recorded and a finger-prick blood sample was collected to measure haemoglobin concentration and to prepare a blood smear. Children who had an axillary temperature ≥ 37.5°C or a history suggestive of malaria and malaria parasitaemia were treated with sulphadoxine-pyrimethamine (SP), the second line drug for management of uncomplicated malaria and allowed the clinician's judgement to prevail. Participants were observed at days 7, 14 and 28 post-treatment to assess clearance of malaria parasites as well as resolution of symptoms. Children who had a negative smear, had a repeat blood smear taken on the following day and were treated with SP if positive for malaria. If a repeat smear was negative and symptoms had subsided, the thrice-weekly examination of temperature and history taking was resumed. If symptoms persisted, daily temperature measurements, blood smears and evaluation by the medical staff continued. Symptomatic patients with negative smears were evaluated for other conditions beside malaria, treated appropriately and followed up. A referral system to the regional hospital was put in place for any child whose condition deteriorated.

### Malaria Surveys

The all-age cohort was surveyed once every eight weeks over the one- year study period. At each survey, axillary temperature and a finger prick blood sample was collected to measure haemoglobin concentration using a calibrated haemocue (HaemoCue GmbH, Germany) and to test for malaria parasites. A medical history was obtained and physical examinations performed; information was also collected on use of a bed net by each participant.

### Examination of blood smears

Thin and thick blood smears were stained with Giemsa after fixing the thin smear with methanol. At least 100 high power fields were examined before a thick smear was declared negative. The number of parasites per micro-litre approximately equals 40 times the number of parasites counted per 200 leukocytes. 10% random selections of smears were independently read by an experienced second reader to ascertain the quality of microscopy. There was a high level of correlation between the microscopists (kappa = 0.94).

### Polymerase Chain Reaction (PCR)

Baseline assessment of chloroquine and SP resistance molecular markers and multiplicity of infection was carried out. Parasite DNA was extracted using the Chelex 100 sodium form (Bio-Rad laboratories) according to the procedure previously described [11]. This DNA was used in primary and secondary (nested) PCR analyses to detect and characterize mutations in the *Plasmodium falciparum *chloroquine resistance transporter (*pfcrt*) and multi-drug resistance *(pfmdr1*) genes. Polymorphisms in the dihydrofolate reductase (*dhfr*) and dihydropteroate synthase (*dhps*) genes were also determined by nested PCR amplifications, and dot blot hybridization as previously described by *Noedl et al *[12] and *Duraisingh et al *[13]. Polymorphic repetitive regions of block 3 of Merozoite Surface Protein-2 (*msp2*) were used for genotyping the *Plasmodium falciparum *isolates using a nested PCR.

The seasonal distribution of parasites within the all-age cohort was determined in 600 participants microscopically positive for malaria. These randomly selected filter paper samples were taken through DNA extraction and analysis, using the chelex method and MSP2 genotyping of *Plasmodium falciparum as *described above. Filtermats were collected from children who were treated with SP for clinical malaria episodes and actively followed up to ascertain the treatment outcome. DNA was extracted from filtermats with corresponding microscopically slide positive samples on the day of treatment (Day Zero), negative on Day 14 and positive on Day 28 after treatment and analysed using the methods developed and routinely used at LSHTM [14,15] described above.

### Estimation of malaria transmission using entomological inoculations rates

CDC light traps were used to collect mosquitoes in rooms of randomly selected households. Trapping was done a day before the malaria survey in each cluster. All houses within the study area were given equal chances of participation. They were selected without replacement until all houses within a stratified area had been considered. Traps were set in at least four houses in each cluster each month. Additional traps were set weekly in rooms of study participants included in the child cohort or the all age cohort ensuring that at least one trapping took place in each month in each of the sixteen (16) clusters and throughout the whole one year period of the study. A total of 664 successful light traps were set in 708 houses. Occupants of each room in which a trap was set were provided with an untreated bed net to be used for the night that the trap was set in his/her room. Traps were hung approximately 1.5 m above the floor at the foot of the bed/mat of the index person. *Anopheline *vectors were morphologically characterized by species using keys [16], stored in 1.5 ml micro-centrifuge tubes enclosed in zip lock plastic bags with silica gel. A maximum of ten vectors of the same species from the same compound was put in a tube. Non-*Anophelines *were discarded after recording numbers caught.

Heads and thoraces of the two major vectors of malaria, *Anopheles gambiae *and *Anopheles funestus*, were checked for the presence of circumsporozoite (CS) antigens of *Plasmodium falciparum *using the sandwiched Enzyme-Linked Immunosorbent Assay (ELISA) as described by Wirtz *et al *[17]. Presence of CSP in the mosquitoes was read at 405 nm wavelength using a micro plate ELISA reader. A cut-off of 0.2 nm absorbance was considered positive after subtraction of an average value from seven negative test mosquitoes. Heads and thoraces of male *Anopheles *vectors were used as the negative test controls. All positive tested mosquitoes were retested to confirm positives.

Sub-samples of *An. gambiae *species were subjected to PCR analysis to identify sibling species within the complex as per the Scott *et al *protocol [18]. Enzyme digestion to differentiate the molecular forms of *An. gambiae s.s*. was performed as per the protocol of Flavia *et al *[19]. The presence of *kdr *alleles associated with knock-down resistance in West Africa was assessed, as described in the Martínez-Torres *et al *protocol [20]. An adjustment to protocols for optimization and to suit local laboratory conditions was done when necessary.

### Sample size calculation

The sample size calculation was based on our assumed estimates of the age-group specific parasite prevalence rates (p) and standard error (s) among children less than 5 years (p = 84%, s = 0.03893), 5-10 years(p = 92%, s = 0.02845), 10-19 years (p = 64%, s = 0.05242), and more than 19 years (p = 51%, s = 0.05242). A minimum number of 140 volunteers was estimated in each age group to represent a cluster. This allowed us to estimate the age specific parasite prevalence rates to within 6 -10% of the true values for the different age groups. The expected precision of the estimates were calculated using the formulae of Bennett *et al *[21].

### Data management and statistical analysis

All data collected in the field or the laboratory were logged for traceability, and then batched for double data entry and processing using Microsoft^® ^Visual FoxPro 6.0. All data management processes were done at the computer laboratory of the Kintampo Health Research Centre. Data analysis was done using StataCorp Stata 9, TX USA. Moving averages and proportions were used to provide descriptions of age and seasonal variation in parasite prevalence, density, infection rates and the proportions of parasite clones that persisted over the period.

### Ethical processes

The study protocol and instruments were reviewed and approved by the Ghana Health Service Ethics Review Committee and London School of Hygiene and Tropical Medicine Ethics Committee. In each community, a meeting was held among community members, their opinion leaders and the investigators to seek approval for the study to be conducted in the community before the study started. Written consent was sought from each adult participant while care-givers gave a written consent on behalf of their children.

## Results

### Prevalence of malaria

**Figure 1:**
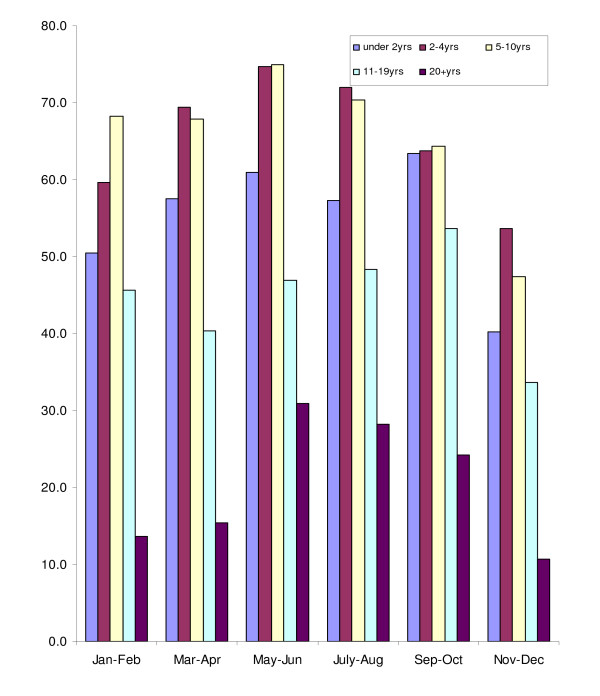
**Parasite prevalence by age group and months of survey**.

**Figure 2:**
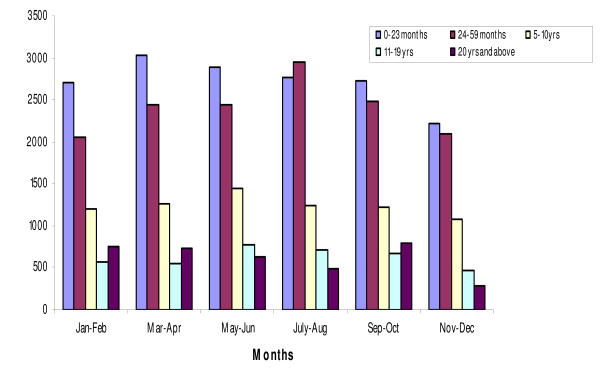
**Geometric Mean Parasite Density by age and month of survey**.

The annual prevalence of malaria over the 12-month period in the all-age cohort was 58% (95% CI: 56.9, 59.4%) and it ranged from 67.3% (95% CI: 64.1, 70.3%) in May-June to 51.9% (95% CI: 49.3, 54.6%) in November-December. The average annual prevalence in children below five years of age was 64% (95% CI: 61.9, 66.0%) and it ranged from 74.3% (95% CI: 68.7, 79.3%) in May-June to 56.8% (95% CI: 53.0, 60.5%) in November-December, a statistically significant difference between the two periods. Parasite prevalence increased with age until about ten years. It was relatively high during the rainy season (May-October) but was high throughout the year (Figure 1). The geometric mean parasite density (GMPD) decreased significantly with age but it remained relatively constant throughout the year for all age groups (Figure 2). There were no statistically significant differences between GMPDs within each age group throughout the year.

### Prevalence of anaemia

**Figure 3:**
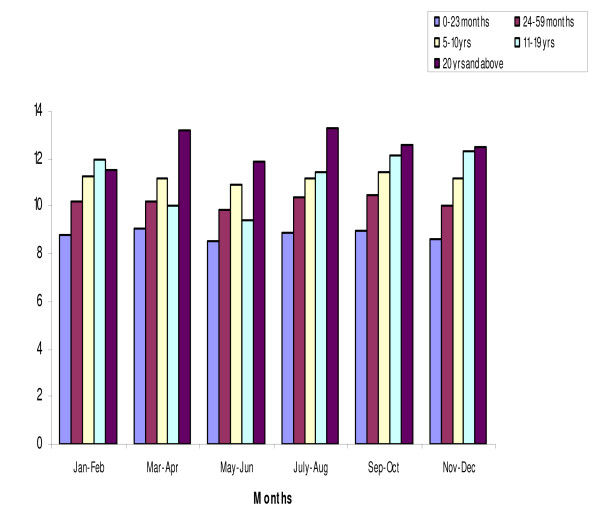
**Mean haemoglobin concentration by age and month of survey**.

**Figure 4:**
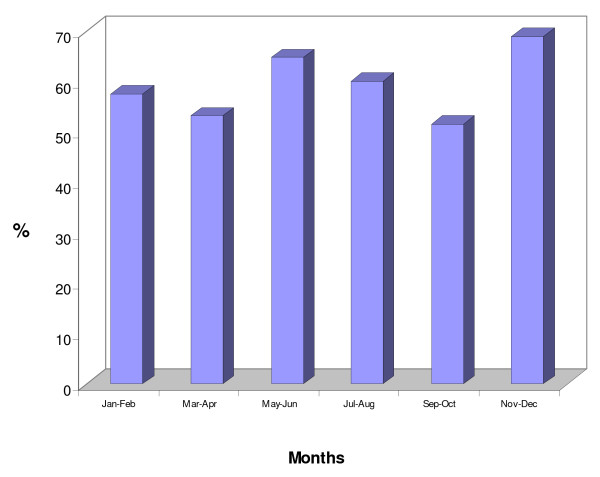
**Haemoglobin concentrations by survey round: % with Hb concentration less than 11 g/dL**.

The mean haemoglobin concentration in the all-age cohort was 10.67 g/dL. It was 10.64 g/dl in males and 10.71 g/dL in females. For the children under-five years of age, the mean was 9.68 g/dL; children <2 year of age had the lowest mean Hb concentration (8.85 g.dL) (Figure 3). Throughout the year, over half of the children less than 10 years of age had a Hb < 11 g/dL (Figure 4). Moderately severe anaemia (Hb <8 g/dL) was highest (12.6%) in November/December, the end of the rainy season.

### Incidence of clinical malaria

The incidence density of clinical malaria defined as any level of parasite density plus reported fever/axillary temperature > 37.5°C among children below five years of age was 7 attacks per child per year. It was 2.3 attacks per year in children below six months of age and peaked in 12-35 month old children (8.6 per child per year). The incidence of malaria with higher parasite density levels was lower than malaria with any parasite density in all age groups (Table 1). The incidence of malaria in children below five years of age was higher in the rainy season compared to the dry season. The prevalence of fever was significantly higher when parasitaemia cut-off levels increased. For children less than two years of age, incidence density with parasite counts below 5,000/uL was 7.8 compared with 9.5 for parasite counts up to 10,000/μL and still higher (11.3) for parasite counts up to 20,000/μL.

**Table 1:** Incidence density of clinical malaria* by age group in under five year-old children

**Age group**	**Episodes of clinical malaria**	**Person Years**	**Incidence density (no. of episodes per child per year)**
**Any Parasitaemia**			
0-6 mth	36	5637	**2.3**
0-11 mth	145	12301	**4.3**
12-23 mth	310	13239	**8.6**
24-35 mth	246	10618	**8.5**
36-47 mth	224	11673	**7.0**
48-59 mth	133	7658	**6.3**
**under-five yrs**	**1058**	**55489**	**7.0**
			

**Parasitaemia >5000/μL**			
0-6 mth	12	5637	**0.8**
0-11 mth	77	12301	**2.3**
12-23 mth	222	13239	**6.1**
24-35 mth	166	10618	**5.7**
36-47 mth	122	11673	**3.8**
48-59 mth	69	7658	**3.3**
**under-five yrs**	**656**	**55489**	**4.3**

			

**Parasitaemia >10000/μL**			
0-6 mth	9	5637	**0.6**
0-11 mth	62	12301	**1.8**
12-23 mth	193	13239	**5.3**
24-35 mth	145	10618	**5.0**
36-47 mth	103	11673	**3.2**
48-59 mth	56	7658	**2.7**
**under-five yrs**	**559**	**55489**	**3.7**

			

**Parasitaemia >20000/μL**			
0-6 mth	7	5637	**0.5**
0-11 mth	45	12301	**1.3**
12-23 mth	146	13239	**4.0**
24-35 mth	108	10618	**3.7**
36-47 mth	80	11673	**2.5**
48-59 mth	46	7658	**2.2**
**under-five yrs**	**425**	**55489**	**2.8**

			

**Parasitaemia >50000/μL**			
0-6 mth	1	5637	**0.1**
0-11 mth	27	12301	**0.8**
12-23 mth	80	13239	**2.2**
24-35 mth	61	10618	**2.1**
36-47 mth	32	11673	**1.0**
48-59 mth	16	7658	**0.8**
**under-five yrs**	**216**	**55489**	**1.4**

* Clinical malaria = parasitemia at defined levels plus either temperature ≥ 37.5°C or reported fever within 48 hours

### *In vivo *drug sensitivity of commonly used anti-malarials in the study area

For SP, the PCR uncorrected treatment failure rate by day 14 increased from 23.3% during a first recorded episode to 40.3% when a child had a fourth or more frequent episode of malaria. Similarly, the treatment failure rate by day 28 increased from 60.8% for first episodes to 79.8% for the fourth or more episodes, suggesting a selection for SP resistant parasites by repeated treatment (Table 2). The adequate clinical and parasitological response for chloroquine on day 14 was 87.7% and for day 28 was 77.1% (Table 3).

**Table 2:** Proportion of children with malaria parasites within 28 days post treatment with sulphadoxine-pyrimethamine at each episode of malaria

**Day of follow up**	**First episode**	**Second episode**	**Third episode**	**Four or more episodes**
	**(N = 288)**	**(N = 237)**	**(N = 183)**	**(N = 382)**
7	24.3	22.8	20.8	29.3
14	23.3	27.0	35.5	40.3
28	60.8	61.2	73.2	79.8

**Table 3:** Proportion of children with malaria parasitaemia within 28 days post treatment with chloroquine

**Day of follow up**	**first episode**
	**(N = 455)**
7	4.2
14	12.3
28	22.9

### Molecular markers for chloroquine and SP resistance and multiplicity of infections

In a sample of 100 children from whom blood samples were collected prior to treatment, the prevalence of mutations at the *pfcrt *and *pfmdr1 *gene loci 76 and 86 were 62.2% and 60.1% respectively. The prevalence of mutations at the *pfdhfr *gene loci 51, 59 and 108 were 51.3%, 66.2% and 60.9% respectively, while the prevalence of mutations at the *pfdhps *gene locus 437 was 74.1%. There were 31 isolates harbouring all three mutant alleles of *pfdhfr *gene. Twenty-five percent of the 31 isolates carried the additional *pfdhps *A437G.

**Figure 5:**
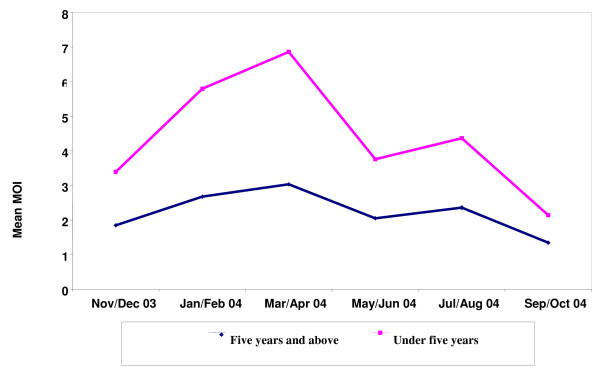
**Mean Multiplicity of Infections by season (MOI)**.

In keeping with the high prevalence of infection, the multiplicity of infections (MOI) was high. The MOI peaked in March-April and this trend was seen in children and adults (Figure 5).

### Malaria vectors and entomological inoculation rate

**Figure 6:**
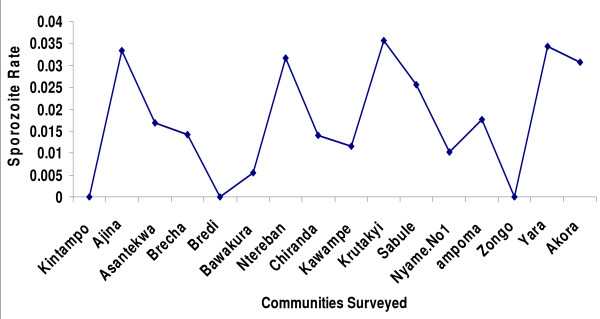
**Mean Sporozoite Rates (SR) in communities in Kintampo**.

**Figure 7:**
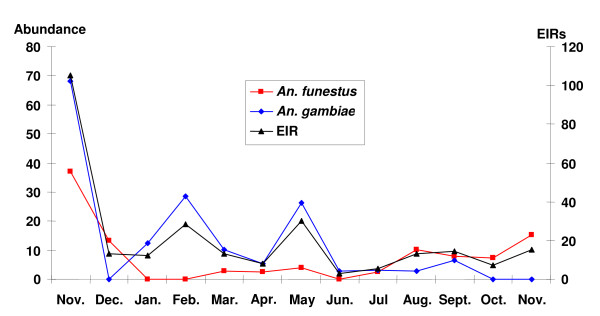
**Monthly vector abundance and EIRs in Kintampo - Nov 2003- Nov 2004**.

A total of 19,835 mosquitoes were collected from light trap catches during the one year study period and comprised *Culex *(51.3%), *Aedes *(1.0%), *Anopheles funestus *(35.2%), *Anopheles gambiae *(10.6%), *Anopheles rufipes *(1.8%) and *Anopheles pharoensis *(0.1%). Among the sample of mosquitoes assayed by CS-ELISA, 1.5% of *An. funestus *(n = 6,542) and 4.7% of *An*. *gambiae *(n = 1,876) were infected with *P. falciparum*. No *An. arabiensis *was found. The estimated annual Entomological Inoculation Rate (EIR) for both *An. gambiae *and *An. funestus *vector species was 269 infective bites per person per year (*ib/p/y*). Infective bites per person per night ranged from 0-13.5 in the communities (Figure 6). There were variations in EIRs by season and between clusters. EIR peaked during the minor wet season, Nov - April (202 *ib/p/y*) and became less during the major wet season, May-Oct (67 *ib/p/y*). Transmission was all-year round, but the two main vectors alternated their peak transmission periods (Figure 7). *An. gambiae *transmission peaked at the end of the rainy season (November to December) whiles *An*. *funestus *transmission peaked during the rains (May to October). This observation reflected in abundance of the vectors as the abundance of *An. gambiae *was very high at the end of rains whiles abundance of *An*. *funestus *was very high during the torrential rains (Figure 7). Nonetheless, *An*. *gambiae *remained the main vector contributing most to transmission despite the lower abundance of this vector population within the survey period. There were marked variations in EIR between communities, depicting the heterogeneity of the micro-ecology in the forest-savanna fringes of Ghana. Monthly fluctuations in vector densities resulted in variations in inoculation rates by season (Figure 7). Inoculation rates were however sustained throughout the year by the two main vectors.

### Species, molecular forms and knock-down resistance (*kdr*)

Only *Anopheles gambiae s.s *were caught and identified as the predominant *Plasmodium falciparum *vector; 55 of the species were randomly selected and tested using molecular techniques. In the wet season samples, DNA was amplified successfully in 22 of which 16 (88.89%) were identified as S-molecular forms, two (11.11%) as M-molecular forms and one (5.56%) as a hybrid (S-M) molecular form. The pattern was similar for 33 samples collected in the dry season where 11 (68.75%) were made up of S-molecular forms, two (12.50%) of the M-molecular forms and three (18.75%) were hybrids (S-M)-molecular forms (Table 4). The frequency of *kdr *resistant genotypes was 0.60 F(R). The genotypes *kdr*^RR ^was observed in the M-molecular form in comparison with the *kdr*^R/S ^genotype which was found in the hybrid (M-S) molecular form. All susceptible genotypes *kdr*^S/S ^were detected in S-molecular forms (Table 4).

**Table 4:** Species, molecular forms and *kdr *genotypes in *An. gambiae *s.s in Kintampo

	**Species**		**Molecular forms**			***Kdr *mutation**			
**Season**	**Ag.**	**Ar.**	**M**	**M/S**	**S**	***kdr***^**RR**^	***kdr***^**R/S**^	***kdr***^**SS**^	**F(R)**
***Wet***	22	0	2	1	16	3	1	1	**0.60**
***Dry***	33	0	2	3	11	3	1	1	

Hardy-Weinberg expected frequencies not significantly different (P < 0.05; df = 1; χ^2 ^= 0.005)

Bed net (treated or untreated) use was low for all age groups. The maximum use of a bed net was found among children less than 5 years of age (25.4%). However, <10% of the community used treated bed nets throughout the year.

## Discussion

The prevalence of malaria is now falling in several parts of Africa [22-25] as the result of scaling up of control interventions such as insecticide-treated bed nets (ITNs) and artemisinin-based combination therapy (ACT) [26,27]. However, this study shows that in the year 2004 the burden of malaria was still very high in the forest/savanna transition of central Ghana. The clinical malaria attack rate and prevalence of malaria in children were very high.

The prevalence of anaemia was very high and consistent with reports in Ghana (84%) [28] and other African countries [29] where malaria was endemic. The high prevalence of anaemia could also be due to malnutrition, hookworm infection, and sickle cell anaemia. However, the contribution of these illnesses to anaemia in a malaria endemic region has been found to be minimal compared malaria [30,31]

The average EIR (269 *ib/p/y*) was also relatively high and transmission was mainly by *Anopheles gambiae *and *Anopheles funestus*. *An arabiensis *had no role in malaria transmission in this area unlike other West-African countries such as Mali [32]. This could be due to the short duration of the dry season in our study area compared to the long duration of dry season in Mali. A similar environmental influence on species survival is demonstrated by the molecular forms of *An. gambiae s.s*. The S-molecular form was predominant in this area with a short dry season contrary the predominantly M-form found in northern Ghana where the dry season is longer [33]. The presence of low frequency of hybrids suggests the possibility of interbreeding and gradual gene flow within the complex.

A number of factors are likely to underlie the reason why the burden of malaria has remained so high in this part of Ghana despite its relatively high level of prosperity and well-functioning health system. Firstly, the ecosystem is well suited to the survival of *An. gambiae *and *An. funestus *with many suitable breeding sites for both species and high humidity persisting throughout the year. Secondly, at the time of this study, attempts at vector control in the area were limited. Coverage with ITNs was low and less than 10% of the community members used ITNs throughout the year and no indoor residual spraying was taking place. This low coverage was similar to most other rural areas of Ghana where ITNs coverage was on the average about 3.5% in 2003 as reported in the Ghana Demographic and Health Survey. Thirdly, at the time of the study, clinical malaria was being treated in government facilities and in the private sector primarily with chloroquine or SP. This study demonstrated a high treatment failure rate with both of these drugs, and a high prevalence of mutations associated with resistance to each of these drugs similar to other reports in Ghana [34].

Following from the results in this study, steps have been taken to strengthen malaria control in the study area. Use of ITNs has been actively promoted through education and free ITNs distribution. The use of ITNs has increased to 60% among children less than 5 years and pregnant women in 2007 (Kintampo Health and Demographic Surveillance Systems updates). Since 2005, the first line treatment for malaria has been changed from chloroquine and SP to the more effective drug combination of artesunate plus amodiaquine, although other anti-malarials are still used in the private sector. It is likely that as a result of these interventions the level of malaria transmission has fallen in the study area since the investigations reported in this paper were carried out and therefore a follow-up survey needs to be undertaken. Nevertheless, in comparison to many other parts of Africa, the transmission of malaria remains high in this zone because the background incidence of malaria in children was very high and there is transmission throughout the year. Thus, the Kintampo area is an appropriate site for the conduct of malaria treatment and vaccines trials. Since the completion of this study a clinical trial of artemether-lumefantrine, amodiaquine and artesunate and artesunate + chlorproguanil dapsone [35] and two phase II trials of the malaria vaccine RTS, S have been carried out.

## Conclusion

Findings from this study have facilitated the design of trials of anti-malarial drugs and vaccines in the study area and led to optimal interpretation of the results from such trials. On the basis of the background epidemiology of malaria described in this paper, the Kintampo Health Research Centre is well-equipped to contribute to the phase 3 trial of the RTS, S vaccine starting in mid-2009 and future treatment and vaccine trials.

## Competing interests

The authors declare that they have no competing interests.

## Authors' contributions

SOA was the lead in writing the proposal with contribution from KPA, DC, JG and BG. SOA, KP, JG and DC were involved in the planning of the study. SOA, KP, MD, SN and DC implemented the study and data collection. MD, DD, DE and AA analysed laboratory specimen. GA, MA and EL contributed to data management. SOA, KPA, MA, GA, EA, DC and BG contributed to data analysis and presentation. SOA, KPA, DC, BG, DE and AA wrote the manuscript. All authors read and approved the final manuscript.
